# Predicting clinical outcome of neuroblastoma patients using an integrative network-based approach

**DOI:** 10.1186/s13062-018-0214-9

**Published:** 2018-06-07

**Authors:** Léon-Charles Tranchevent, Petr V. Nazarov, Tony Kaoma, Georges P. Schmartz, Arnaud Muller, Sang-Yoon Kim, Jagath C. Rajapakse, Francisco Azuaje

**Affiliations:** 1 Proteome and Genome Research Unit, Department of Oncology, Luxembourg Institute of Health, 1A-B, rue Thomas Edison, Strassen, L-1445 Luxembourg; 2 Bioinformatics bachelor program, Universität des Saarlandes, Saarbrücken, Germany; 3 Bioinformatics Research Center, School of Computer Science and Engineering, Nanyang Technological University, Singapore, Singapore

**Keywords:** Biological networks, Network-based methods, Network topology

## Abstract

**Background:**

One of the main current challenges in computational biology is to make sense of the huge amounts of multidimensional experimental data that are being produced. For instance, large cohorts of patients are often screened using different high-throughput technologies, effectively producing multiple patient-specific molecular profiles for hundreds or thousands of patients.

**Results:**

We propose and implement a network-based method that integrates such patient omics data into Patient Similarity Networks. Topological features derived from these networks were then used to predict relevant clinical features. As part of the 2017 CAMDA challenge, we have successfully applied this strategy to a neuroblastoma dataset, consisting of genomic and transcriptomic data. In particular, we observe that models built on our network-based approach perform at least as well as state of the art models. We furthermore explore the effectiveness of various topological features and observe, for instance, that redundant centrality metrics can be combined to build more powerful models.

**Conclusion:**

We demonstrate that the networks inferred from omics data contain clinically relevant information and that patient clinical outcomes can be predicted using only network topological data.

**Reviewers:**

This article was reviewed by Yang-Yu Liu, Tomislav Smuc and Isabel Nepomuceno.

**Electronic supplementary material:**

The online version of this article (10.1186/s13062-018-0214-9) contains supplementary material, which is available to authorized users.

## Background

In the last decade, high-throughput technologies have been massively used to study various diseases in order to decipher the underlying biological mechanisms and to propose novel therapeutic strategies. Initiatives such as The Cancer Genome Atlas have produced and made publicly available a huge amount of omics data from thousands of human samples. These data often correspond to measurements of different biological entities (*e.g.*, transcripts, proteins), represent various views on the same entity (*e.g.*, genetic, epigenetic) and are obtained through different technologies (*e.g.*, microarray, RNA-sequencing). This diversity has motivated the use of integrative strategies that can make sense of these complementary and sometimes contradictory data. Such integrative strategies have, for instance, been used to define distinct molecular classes of lower-grade gliomas, which exhibit similar pathway perturbations [1].

Biological data are often represented as networks, where nodes represent biologically relevant entities (typically genes or proteins) and edges represent relationships between these entities (*e.g.*, regulation, interaction). Network-based methods can then be used, for instance, to define smaller modules within a larger network, or to understand how a biological signal is processed by a network, or to identify key nodes with respect to a biological process of interest. As an example, such network-based approaches have been used to build brain region-specific networks from patient expression profiles and to prioritize genes and gene sets with respect to Alzheimer’s disease traits [2]. It is also possible to obtain relevant predictive models by relying on the network topological information, instead of the raw data. An example of such method is Mashup, an approach that summarizes topological information from protein-protein networks to predict functional annotations or genetic interactions, yielding comparable or often even better performance than other state of the art methods [3].

Although most biological networks represent gene or protein networks, it is often relevant to represent the data as Patient Similarity Networks (PSN). In these networks, nodes represent patients and edges represent similarities between the patients’ profiles. These networks can be used to group patients and to associate these groups with distinct clinical features. It was observed for instance that, within a network obtained by integrating multiple omics data, cancer patient clusters had different clinical outcomes, including different overall survival [4]. Similarly, a network topology-based analysis of diabetes patient genotypes revealed that patients can be clustered into three groups and that these groups have distinct clinical features, including different comorbidities [5].

In the current study, we hypothesize that clinically relevant information is encoded within PSN built from omics data. To investigate whether we can use this topological information to predict patient clinical outcome, we analyze a neuroblastoma dataset in the context of the CAMDA 2017 conference [6]. This dataset contains gene expression data, genotype data and clinical descriptors. In a previous analysis, patient classifiers were built from the gene expression data and were used to predict several clinical outcomes [7].

Our approach is however different since we transform the omics data into networks and then train patient classifiers with network topological data, instead of training the classifiers directly with omics data. Our results indicate that the performance of classifiers trained with topological data is at least comparable to the performance of the models built on the omics data directly and in some cases better. Altogether, our network-based approach represents therefore a novel and complementary strategy to analyze and integrate large collections of omics data.

## Results

**Fig. 1:**
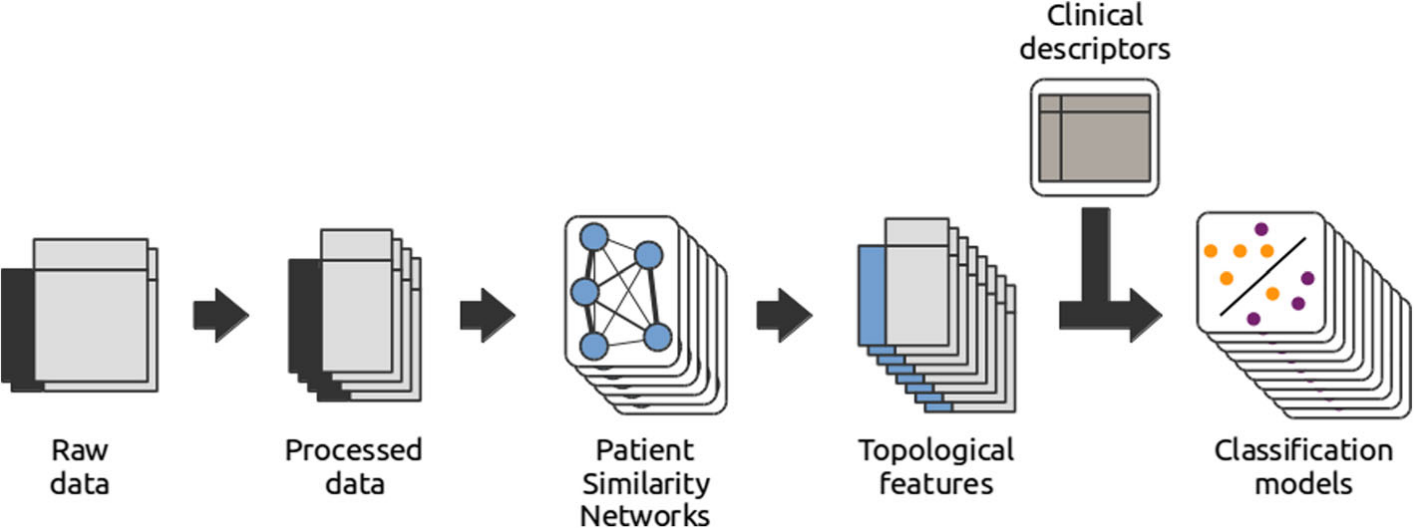
Workflow of our network-based method. The raw omics data are first processed into data matrices by applying dimensionality reduction. The selected omics features are then used to infer Patient Similarity Networks (PSN), from which topological features are extracted. These network topological features are then used to build classification models, with classes defined according to the binary clinical descriptors

We propose a network-based method to integrate omics data, which relies on the topological properties of networks generated from the omics data (see Fig. 1 and “Methods”). More precisely, relevant features are first identified from the omics data and then used to create patient similarity networks. Second, four sets of network topological features are extracted, including (i) centrality metrics, (ii) *node2vec* features, (iii) diffusion features and (iv) modularity features. These topological features are then integrated into patient classification models (see “Methods”). The classes are defined using binary clinical descriptors and the models, trained on half of the samples, are used to predict the values of these clinical descriptors for the other half of the samples. In the context of one of the CAMDA 2017 challenges, we have applied our strategy to a neuroblastoma dataset that combines genomic, transcriptomic and clinical data from 498 patients. In the following sections, we describe the classification performance under different settings to investigate the effectiveness of the proposed strategy on two cohorts of respectively 498 and 142 patients (Table 1).

**Table 1:** Summary of the experiments described in the manuscript together with their global settings

Tag	Cohort	Model integration ^*a*^	Feature sets	Data sources
Classical ^*b*^	Both	No	-	All ^*c*^
Topological ^*b*^	Both	Yes	All	All ^*c*^
Integrated ^*b*^	Both	Yes	All	All ^*c*^
Centrality	Both	No	Centralities (all)	All ^*c*^
Single centrality	Both	No	Centralities (one)	All ^*c*^
node2vec	Both	No	node2vec	All ^*c*^
Diffusion	Both	No	Diffusion	All ^*c*^
Modularity	Both	No	Modularities	All ^*c*^
Transcriptomic (microarray)	Both	No	All	Transcriptomic (microarray)
Transcriptomic (RNA-seq)	Both	No	All	Transcriptomic (RNA-seq)
Transcriptomic (both)	Small ^*d*^	No	All	Transcriptomic (both)
Genomic (aCGH)	Small	No	All	Genomic
Fused	Both	Yes	All	All ^*c*^

For the parameters that are not mentioned (*e.g.*, dimension reduction strategy, network inference method, classification algorithm), the experiments are repeated for all possible values. ^*a*^Integration with weighted voting scheme. ^*b*^An equivalent tag for these models on the small cohort is *All three sources*. ^*c*^This means two on the large cohort and three on the small cohort. ^*d*^On the large cohort, it is equivalent to the topological model

**Fig. 2:**
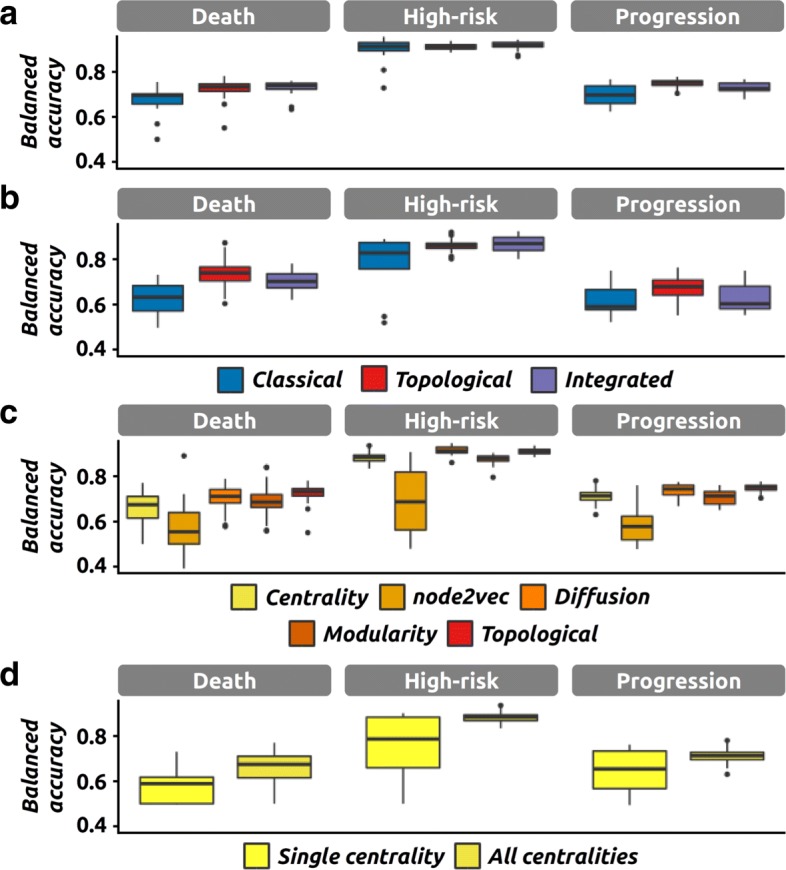
Performance of the network-based method and its components. The performance (*i.e.*, balanced accuracy) of classification models in various settings, and for the three clinical endpoints of interest. **a** Performance of classical, topological and integrated models on the large cohort (498 samples). **b** Performance of classical, topological and integrated models on the small cohort (142 samples). **c** Performance of models using only one of the four feature sets at once (Centrality, node2vec, diffusion and modularity) or all of them (topological, as in **a**). Results were obtained on the large cohort. **d** Performance of models using a single centrality metric or all centrality metrics at once. Results were obtained on the large cohort

We have first compared the performance of the classification models when inputted with omics data (*hereinafter* classical) or with the network derived features (*hereinafter* topological), regardless of the other parameters. Our results indicate that both strategies behave similarly across the three clinical endpoints considered (Fig. 2a-b and Additional file 1: Figure S1) with ‘*Disease progression*’ and ‘*Death from disease*’ being more difficult to predict than ‘*High-risk*’. The topological strategy however performs significantly better than the classical strategy for five of the six comparisons (three endpoints and two cohorts - Additional file 1: Table S1), and the average gain in balanced accuracy ranges from 5% to 12% (excluding the non-significant comparison).

We then defined a global classification model that combines the topological and classical approaches to investigate their complementary (*hereinafter* integrated). More precisely, their individual predictions are integrated using a weighted voting scheme (see “Methods”). The results indicate that the integrated models perform significantly better than the classical models (in four out of six comparisons). However, they are most often associated with smaller accuracy gains (between 3% and 8%, excluding the two non-significant comparisons). We do not observe any signficiant difference between topological and integrated models and the accuracy gain is always lower than 5% (Fig. 2a-b and Additional file 1: Table S1).

Upon a closer investigation, we can also observe differences between the four topological feature sets when used individually (Fig. 2c). In particular, the best models are the ones using centrality metrics and diffusion features, whereas the *node2vec* features are associated with lower accuracies in general.

We also performed a comparison of the individual centrality metrics. We first observe that using all twelve metrics give better models than using any metric in isolation, which was observed for all clinical endpoints on the large cohort (*Δ*_*bACC*_ between 7% and 12%, Fig. 2d, and Additional file 1: Table S1). For the small cohort, we observe a similar trend although it is not significant. A closer look at the performance of the models based on a single centrality metric reveals differences, with metrics associated with high average performance (*e.g.*, eigenvector centrality, hits) or low average performance respectively (*e.g.*, load, current-flow betweenness) (see Additional file 1: Figure S2). Another key observation is that the iterative versions of weighted degree and local clustering coefficient are associated with lower average performance than their non-iterative counterparts.

**Fig. 3:**
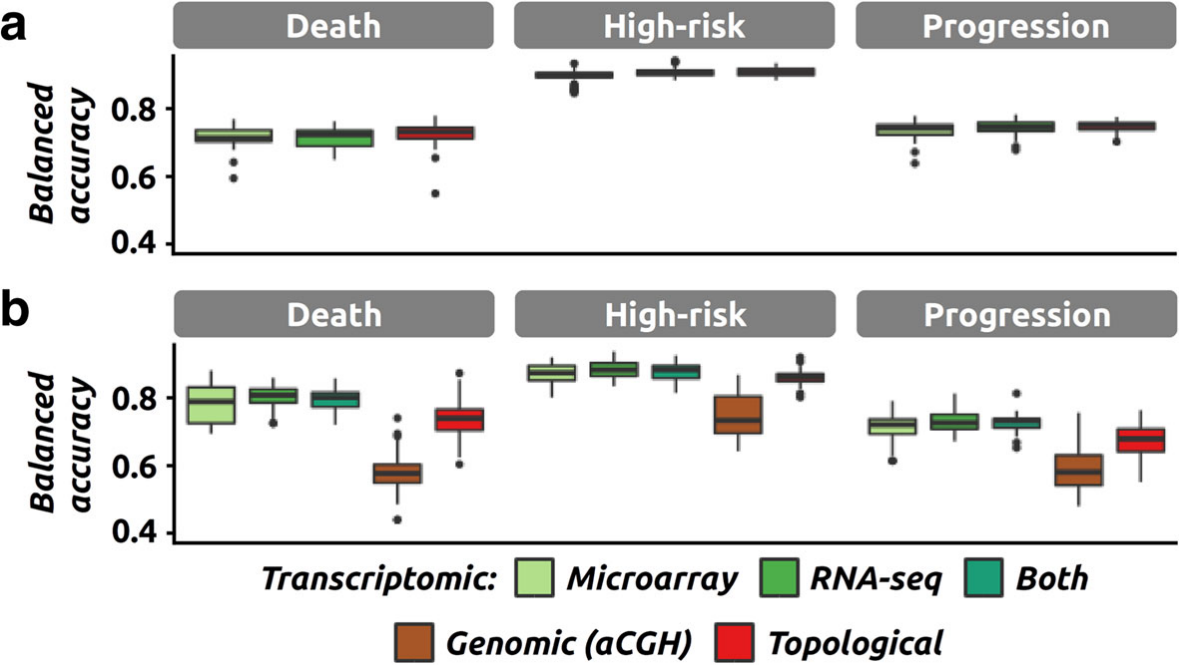
Impact of the data sources on the performance. The performance (*i.e.*, balanced accuracy) of classification models in various settings, and for the three clinical endpoints of interest. **a** Performance of the topological models relying only on a single transcriptomic data source (greens), or on both sources (red, equivalent to the topological model presented in Fig. 2a). Results were obtained on the large cohort. **b** Same as **a** but on the small cohort. Performance of topological models using one (greens and maroon), two (dark green, only transcriptomic) or three data sources (red, equivalent to the topological model presented in Fig. 2a)

We then investigated the power of individual data sources among the three at our disposal (one genomic and two transcriptomic, microarray and RNA-seq). Regardless of the cohort, we can observe very similar performance between models using either only the microarray data, only the RNA-seq data or both (Additional file 1: Table S2, Fig. 3a-b for topological models and Additional file 1: Figure S3 for classical models). In order to measure the influence of having genomic data, we compared models including and excluding the aCGH data using only the 142 samples associated with genomic data. Using topological models, we observe a surprising decrease in performance when including genomic data, which was observed for two of the three clinical endpoints (Fig. 3b and Additional file 1: Table S2). We observe a similar trend for classical models although none of the comparisons are significant (Additional file 1: Table S2 and Figure S3). This observation was further confirmed by the significantly lower accuracy of topological models built solely on genomic data with respect to topological models using the other data sources (Additional file 1: Table S2, *Δ*_*bACC*_ between 12% and 23%).

In our approach, multiple networks are derived in parallel and their topological features are then combined at the modeling stage (late integration scheme). An alternative strategy is to integrate the data at the network level (intermediate integration scheme) and build models from the fused network features (“Methods”). We observe that these two strategies are associated with similar performance (Additional file 1: Table S1 and Figure S4) across the three endpoints and two cohorts (*Δ*_*bACC*_≤3%).

Similarly, we observe no impact on the performance of the data processing parameters (dimensionality reduction and network inference strategies), and neither of the various classification algorithms and their optimization (Additional file 1: Figure S5).

## Discussion

We propose a novel method to analyze omics data through the generation of patient similarity networks and their associated topological features. We have analyzed omics data from neuroblastoma patients and integrated the derived topological features into classification models that can be used to predict patient clinical outcomes. This strategy is novel since it does not use the omics data directly but rather features derived from such datasets. We have demonstrated that our approach outperforms the state of the art method on a neuroblastoma dataset, for all clinical endpoints (Fig. 2a-b).

In agreement with previous studies, we observe that clinical features such as ‘*Death from disease*’ and ‘*Disease progression*’ are more difficult to predict than ‘*Gender*’ and ‘*High-risk*’ [7]. Unfortunately, these more complex clinical outcomes are the ones that are directly of interest for clinicians. Similarly to previous analyses of these data, we present models whose performance for these complex outcomes is still ameliorable (bACC 69-75% - MCC 0.38-0.55). This was expected since our omics-based models are directly inspired by the previously described models [7].

One difference between the topological and classical approaches is the number of features used for classification. In general topological models tend to have less features (1,301 on average per dataset when combining all four feature sets) when compared to the classical models (2,164 and 2,191 for the transcriptomic datasets and 1,933 for the genomic dataset after dimension reduction). In that respect, it is therefore interesting to notice that there is no major difference in terms of performance (Additional file 1: Table S1, accuracy gain < 2%) between models using centrality metrics only (12 features per transcriptomic dataset) and models using the transcriptomic data (at least 2,164 features per dataset).

Another interesting observation is that the *node2vec* feature set sometimes produces less powerful models for all three clinical endpoints considered, even though the tool was run with two distinct configurations, with the objective of building feature vectors that would represent two complementary random walk explorations (local versus global) [8]. A preliminary analysis revealed that the feature vectors built by *node2vec* are not very stable when one parameter is slightly modified (see Additional file 2). This has potentially a significant impact on the effectiveness of the feature vectors. However, these observations are only preliminary and more detailed analyses are required to fully understand how we can best make use of these features.

With a similar objective, we also investigated the predictive power of individual centrality metrics. We first observed that using all centrality metrics gives better results than using any single centrality alone (Fig. 2d). In addition, we observe differences among the centrality metrics. For instance, metrics such as eigenvector centrality and local clustering coefficient are associated with average performance values among the highest. At the other end of the spectrum, load centrality seems to be completely useless in our case, and the current-flow betweenness only does a little bit better. Interestingly, the iterative versions of weighted degree and local clustering coefficient give significantly worse results than the corresponding non-iterative metrics. This is somehow in disagreement with the recent observation that computing centralities iteratively can produce a more useful metric [9]. This observation is however preliminary since we have only implemented and tested two iterative metrics.

Genomic data have been produced recently to complement the already available transcriptomic data, but only for a subset of patients (145 out of 498). One of the main targets of the CAMDA challenge was to build models that would take advantage of these genomic data. Unfortunately, we were not able to improve the models by using aCGH data. On the contrary, we observe a decrease in performance. We noticed that similar observations have been made by other CAMDA participants when using the raw genomic data [10, 11]. We can hypothesize that the significant reduction in sample size is making the classification task harder, in particular because we only have 70 samples left for training. Another possible explanation is that the subset of patients for which genomic data have been generated has not been selected randomly but rather to target specifically patients associated with unknown mechanisms or unforeseen outcomes. This is compatible with the observation that the drop in performance is also observed when only transcriptomic data are used (for two of the three endpoints). Lastly, we can also not rule out that the rather complex design of the aCGH experiment (different laboratories, different techniques, annotation errors) is impacting our analysis [12]. However, larger genomic datasets would be needed to investigate this issue further.

Our attempts to integrate the predictions of both classical and topological models through a weighted voting scheme did not result in higher performance in general (Fig. 2a-b). This lack of improvement can be explained by the fact that the individual predictions are most of the time highly correlated; thus any combination can only result in a modest improvement. However, on the large cohort, we can observe that there is less variation in performance when different classification models are combined. This is because our voting scheme is still efficient when the poorly performing models are in minority. When there is no a priori knowledge about which model might be the best, it might therefore be relevant to integrate several features (both omics and topological) in order to identify a consensus.

The dimensionality reduction strategy seems to have a rather modest impact on the performance (Additional file 1: Figure S5). Surprisingly, specific features (*i.e.*, features that were selected specifically to discriminate classes, using for instance a Wilcoxon test) do not exhibit a better median performance when building models for the corresponding classes. Altogether, this reveals that although specific features should be the preferred option, when computing power is limited or when aiming for generic models (that can be used to predict yet unknown clinical outcomes), other strategies might be almost equally powerful.

The network-based and classical models also differ by their interpretation. Per definition, the features of the topological models do not represent static biological entities (genes / transcripts) but rather the structure and the dynamics of the entire patient network. Therefore, a predictive feature is not a classical biomarker (*i.e.*, a gene) but rather a metric representing how one sample of interest correlates with other samples. As such, we believe that a network-based representation offers the user an alternative interpretation of predictions based on the analysis or the visualization of related diagnostic cases. In the end, we do not consider the proposed strategy as a substitute of existing methods but rather as a way to augment and complement them.

## Conclusions

In this study, we explore the hypothesis that clinically networks encode clinically relevant information through their structure. In the context of the CAMDA 2017 challenge, we analyze omics data from neuroblastoma patients by representing the data as Patient Similarity Networks. Topological features extracted from these networks are then used to build models that classify patients into clinically relevant categories. Our results indicate that our network-based models outperform state of the art models. We observe however that the gain in accuracy is moderate and that the proposed models can still be improved. It would be interesting for instance to investigate the usefulness of other dimension reduction, network inference, and data integration techniques, as well as the inclusion of other topological features. In addition, we are planing on investigating the usefulness of the proposed method on different datasets, covering different disease types.

## Methods

### Data preparation

The transcriptomic datasets were collected on the 28th of February 2017 from GEO [13] using the following identifiers: GSE49710 (microarray) and GSE62564 (RNA-seq). The aCGH data were collected on the same day from the Boku website [14] as specified in the CAMDA guidelines [6].

The clinical descriptors were extracted from the above mentioned datasets and uniformized manually to keep only three clinical descriptors (death from disease, high-risk and disease progression). All clinical descriptors are binary and are available for all 498 patients. The original data have been described previously [7, 15–18]. The two expression datasets contain pre-processed profiles for 498 samples, corresponding to 498 patients.

For aCGH, we extracted the 185 samples, corresponding to 145 patients for which we also had expression data. To account for the fact that the aCGH data were produced using different technologies, the profiles were filtered to keep only the genomic features that are shared by all platforms. In addition, the signal for 30 samples was inverted to correct potential annotation errors (see Additional file 3). Since the aCGH data were produced by different laboratories and using different arrays, the data was further normalized to correct for the potential lab, platform and batch effects. After this processing, we kept 181 samples for 142 patients, replicates were averaged. More details about the genomic data preprocessing can be found in Additional file 3.

Because not all patients were associated with genomic data, we defined two patient cohorts, tagged large and small, and corresponding respectively to all patients with transcriptomic data available (498) and to all patients with both data type available (142).

For all datasets, features with at least one missing point were dropped prior to the network inference step. We then applied two dimension reduction strategies. Our first strategy is based on a Wilcoxon analysis that identifies the features that behave differently between sample groups that are defined using the binary clinical endpoints. The selected features are therefore specific for each clinical endpoint. Briefly, for each clinical endpoint of interest, we either kept all significant features (with *p* < 0.05), or the top 10% features, regardless of their significance. Our second strategy aims at identifying the features that vary the most. Contrary to the first strategy, the features are thus selected independently of the clinical endpoints. In this case, we either kept the 10% most varying features, or the PCA based pseudo-features that explained more than 90% of the variance. In addition, all analyses were also performed with the complete dataset, *i.e.*, without dimensionality reduction.

### Network inference

After dimensionality reduction, each data matrix was then used independently to infer Patient Similarity Networks (PSN). First, the Pearson correlation coefficients between all patient pairs were computed. Then, these correlation coefficients were normalized and rescaled to represent positive edge weights using Weighted Correlation Network Analysis (WGCNA), which enforces scale-freeness of the associated network [19]. The approach is summarized by

1 $$ w_{a,b} = \left(\frac{c_{a,b} - \min(C)}{\max(C) - \min(C)} \right)^{\beta}, ~  $$

with *w*_*a,b*_ the edge weight between the nodes representing the patients *a* and *b*, *c*_*a,b*_ the correlation between the molecular profiles of patients *a* and *b*, *C* the set of all correlations (between all pairs) and *β* the parameter that controls the scale-freeness of the network. As recommended previously, we used the smallest *β* that gives a truncated scale-free index of at least 90% (for our networks, *β*∈{2,4,6,8,10,12}, tag = WGCNA) [2]. Alternatively, and as a control, the normalized coefficients (*β*=1) were also used to infer additional networks (tag = correl).

Network fusion was achieved using SNF [4] with the number of neighbours *K* and the number of iterations *T* set to 10 and 50 respectively, after preliminary testing using a grid search (*K*∈[10;30] and *T*∈[10;100], data not shown).

### Network topology

For each network, we then computed twelve centrality metrics: weighted degree, closeness centrality, current-flow closeness centrality, current-flow betweenness centrality, eigen vector centrality, Katz centrality, hit centrality, page-rank centrality, load centrality, local clustering coefficient, iterative weighted degree and iterative local clustering coefficient. Iterative metrics were computed according to a previous definition [9]. Briefly, at each iteration, only the value for the most central node is kept (highest centrality), this node is then removed from the network and the procedure is repeated until all nodes have been removed. All centrality features were then individually standardized to a zero mean and a unit standard deviation. Each node is then represented by twelve centrality features.

Modularity features were extracted using two network clustering algorithms. First, spectral clustering and Stochastic Block Models (SBM) algorithms were used to split networks into modules. In both cases, the optimal number of modules was defined using dedicated methods from the respective packages. In most cases, several module partitions were identified as optimal and were therefore kept to build the features. Module membership was then transformed into binary features. Each node is then represented by $ \sum _{s \in S}k_{s}$ features, with *S* the set of optimal module repartitions, and *k*_*s*_ the number of modules for repartition *s*.

Each network was also inputted into the tool *node2vec* to produce a feature vector for each node. These vectors are accurate representations of the behaviour of a random walk on the network. In theory, they can be used to reconstruct random walks [8] but in practice, we used them as features for our classification problem. The tool *node2vec* was run twice with different settings, to take advantage of the ability of the tool to favor either local or distant exploration. The default parameters were used (including *d*=128 for the size of the outputted feature vector), except for the return parameter *p* and the inout parameter *q* (which control respectively the probability to return to the previous node and to move to distant node when exploring the networks) that are respectively set to 1 and 4 for the first run; and 4 and 1 for the second run. The final vector was then obtained by concatenating the results of the two runs. Each node is then represented by 256 *node2vec* features.

Last, a diffusion strategy was used to build another feature vector for each node [20]. Each feature was derived from a single diffusion process and 1,000 features were computed in total. For each diffusion, 10% of the nodes were randomly selected and associated with a positive signal (value set to 1), while the remaining nodes were associated with a null signal (value set to 0). The signal for all nodes after diffusion was used to build the feature vector corresponding to that diffusion. As a results, each node is associated with 1,000 diffusion features.

These four feature sets (centrality, modularity, *node2vec*, diffusion) were then considered as features that can be used for classification.

### Classification algorithms

Class definitions have been extracted from the binary clinical descriptors. To facilitate comparisons with previous or alternative approaches, we have used the same train and test stratified split that was previously used [7]. Several classification algorithms were investigated, including Linear Discriminant Analysis (LDA), Random Forest (RF) and Support Vector Machine (SVM). Similarly to the original study, we performed a ten times five fold cross-validation on the training set to get an unbiased estimate of performance. Unless otherwise indicated, the default parameters of the algorithms have been used. For SVM optimization, the parameters *α* and *γ* were optimized via a grid search (*α*=2^2*p*^ and *γ*=2^2*p*^ with *p*∈[−4,−2,−1,0,1,2,4]).

In addition, we considered several classification scenarios by varying the number of data sources, networks and feature sets used. As a control, we also built classifiers using the original omics data (without any network inference). The performance of the classifiers on the test data was estimated using the classification accuracy (ACC), balanced accuracy (bACC) and the Matthews Correlation Coefficient (MCC), similarly to a previous analysis of these data [7].

Predictions extracted from several classification models were then combined using a weighted voting scheme. For instance, each topological model was obtained by combining four classification models (one per feature set). Using the same strategy, each integrated model was obtained by combining a classical model with the corresponding topological model. In both cases, the weights were proportional to the performance of the respective models and were normalized as to sum up to one. A score for each patient was produced by combining the predictions of the individual models. This score was further refined into a binary prediction (using 0.5 as a threshold).

We have employed t-tests for pairwise comparisons and one way ANOVA followed by post-hoc Tukey tests when comparing more than two groups. We only consider the difference as statistically significant when the *p*-value is below 0.01. In addition to *p*-values, we also report the accuracy gain, computed as the difference between the averages of the two groups and labeled *Δ*_*bACC*_.

### Implementation

We developed C++, R and python scripts for the data preparation, network inference, network topology computation and classification analyses. In particular, the batch effect correction was performed using the R package sva. The network inference and centrality computation was based on the python packages networkx and scipy and on the C library igraph. The modularity analysis was performed using the python package graph-tool and the R package SNFtool. The latter was also used for network fusion. The diffusion was performed using the R packages diffuStats and igraph. The classification process relied on R packages randomForest and e1071 and python package sklearn. Statistical tests were run in R.

## Reviewers’ comments

### Reviewer’s report 1: Yang-Yu Liu

**Reviewer comment:** Since the topology-based classification does NOT drastically outperform the classical omics-based classification, what’s the strong motivation of doing topology-based classification? In particular, they can briefly compare and discuss the interpretability of the two classification models.

Author’s response: *The nature of the model features is different between topology-based and omics-based approaches. Per definition, the features of the topological-based models do not represent static biological entities (genes / transcripts) but rather represent the structure and the dynamics of the entire patient network. This means that the interpretation will be different as well. For a new sample, the prediction could either be based on a set of potential biomarkers (omics-based methods), or on the correlation of the entire sample with other samples (network-based methods). As such, we believe that a network-based representation offers the user an alternative interpretation of predictions based on the analysis or the visualization of related diagnostic cases. In the end, we do not consider our strategy as a substitute of existing methods but rather as a way to augment and complement them. We have updated the text to highlight these differences. In addition, the topological approach now outperforms the classical approach (owing to the implementation of the suggestions from the three reviewers).*

**Reviewer comment:** The authors construct the PSN for each data type. But in Ref. [4], an interest method has been proposed to aggregate (or fuse) PSNs from different data types. Will the aggregated PSN offer better topological features for the classification purpose?

Author’s response: *We thank the reviewer for the suggestion. We have extended our analysis to fused networks and have updated the text accordingly. As suggested, fused networks have been created using SNF (as described in [4]), and by fusing either two or three networks (corresponding to either two and three data sources). Briefly, the results indicate that the fused networks offer useful topological features. However, we can also observe that these models do not outperform the models based on the features extracted from individual networks. The manuscript has been updated to include details about the fusion method and to describe and discuss the results obtained on fused networks.*

**Reviewer comment:** In Fig. 3B and Figure S4, the authors showed a very surprising result that by including genomic data, both topological and classical classification models perform worse. They can offer an explanation.

Author’s response: *This observation was discussed in the previous version (page 6, line 33 and page 7, lines 1-6). We hypothesized that the rather low number of samples made the classification task harder, an hypothesis that other CAMDA participants have also made (Francescatto et al., reference [10] in the revised version). In addition, we also hypothesize that the genomic data was only produced for a non random selection of patients, namely the ones with unexpected disease developments, which would likely make the problem harder when working only on this subset. Last, we can also not rule out that the rather complex design of the aCGH experiment poses a hurdle, given the heavy pre-processing that was required prior to modeling (fully described in Additional file 2). This problem was also reported by other CAMDA participants (Suo et al., reference [12] in the revised version). We have updated the manuscript to mention that other CAMDA participants also reported surprisingly low performance when using the genomic data ([10, 11]). We also observed that most participants did not actually use the genomic data at all but it is unknown whether this decision was based on the lack of added value of these data.*

**Reviewer comment:** In Figs. 2, and 3, when the authors compare the performances of the different classification models, they can show the p-value to indicate any significant difference.

Author’s response: *We have employed ANOVA followed by a post-hoc Tukey test to determine significant differences. However, we do not only rely on these tests to discuss the performance and also report the effect size (i.e., difference in average balanced accuracy that we termed accuracy gain). Our motivation is that given the large numbers of models, it is possible to observe a significant difference between two groups (with say a p-value below 1e-7) though the gain in accuracy is unlikely to represent a significant improvement (say 1% or 2%).*

**Reviewer comment:** All the topological features considered here are node-based. How about edge-based centrality metrics (e.g., edge betweenness), and global topological feature (e.g., global clustering coefficient, modularity, etc.)?

Author’s response: *We thank the reviewer for the suggestion. The extracted topological features are then used to classify nodes and therefore node-based features are required. In order to test edge-based metrics, we summarized edge-based metrics (e.g., edge betweenness) at the node level (e.g., by taking the average). We then observed that such features were redundant with existing node-based metrics. For instance, summarized edge betweenness is perfectly correlated with node betweenness, as expected intuitively. We therefore decided to discard them prior to model building. Some global topological features (e.g., global clustering coefficient) are network-based metrics. Therefore, they cannot be used for node classification since all nodes would be associated with the same value. Other global features are however very relevant. We have therefore extended our analysis by including an extra feature set that represents the modularity of the nodes (based on network clustering). More precisely, each modularity feature contains binary values and corresponds to a network module (either a node belongs to a module or it does not). Briefly, the main conclusion is that modularity features are also suitable on their own to build predictive models. They have therefore been integrated with the other topological feature sets and we have updated the manuscript accordingly.*

**Reviewer comment:** Page 5, Line 22, ‘than’ → ‘as’.

Author’s response: *Thank you. This has been corrected.*

### Reviewer’s report 2: Tomislav Smuc

**Reviewer comment:** Focus on one dataset/problem: The work is focused on computational methodology, rather than on biological problem. In that respect having results from studying only one problem (dataset) somewhat limits interpretation, insights gained and impact made, in general.

Author’s response: *We focused on a single dataset because we wanted to describe our solution to one of the CAMDA 2017 challenges, which was about a single neuroblastoma dataset. However, we also agree that additional studies are necessary in order to investigate the usefulness of such strategies on other problems. We have therefore updated the text accordingly.*

**Reviewer comment:** General structure and settings for the computational experiments are clear, but there seem to be a number of unclear or missing information when going into details, which are detailed in my recommendations. I endorse the publication - but I strongly suggest the authors to first try to improve their manuscript along the recommendations.

Author’s response: *We thank the reviewer for highlighting the sections with unclear or missing information. Detailed replies are available below. We hope that our revised manuscript reads better.*

**Reviewer comment:** Comparison with previous results: There is no explicit comparison between the authors’ results, and those obtained by other groups (or best results) - with some other methodology, obtained at CAMDA 2017 challenge. What is the reason for this?

Author’s response: *We have revised the discussion to include more details about the comparison to the state of the art methods (previous analysis of the same dataset [7]). It is important to bear in mind that our omics-based models were inspired by this study and it is therefore not surprising that the performance of our omics-based models is very much in agreement with the performance of the models described in the original study. We have now mentioned the work by other CAMDA participants when discussing the poor performance associated with genomic data. To our knowledge, no other research group has developed similar predictive models that ours could be compared to (most papers in the proceedings describe Cox models and Kaplan-Meyer curves, which cannot readily be used for comparison to our classification models).*

**Table 2:** Results of the Chi-squared tests on the clinical descriptors of the CAMDA 2017 neuroblastoma dataset

	Gender	Age	MYCN	Risk	Stage	Prog	Death
Gender		1	1	1	1	1	1
Age	0.61		5.3e-4	8.8e-28	1.6e-19	3.6e-7	4.8e-11
MYCN	0.50	2.5e-5		3.2e-44	7.4e-11	2.3e-8	8.2e-17
Risk	0.09	4.2e-29	1.5e-45		1.7e-57	3.4e-25	1.6e-34
Stage	0.43	7.7e-21	3.5e-12	8.2e-59		4.2e-21	1.9e-21
Prog	0.58	1.7e-8	1.1e-9	1.6e-26	2.0e-22		1.2e-49
Death	0.37	2.3e-12	3.9e-18	7.5e-36	9.0e-23	5.5e-51	

Results are presented for all pairwise comparisons with Bonferroni corrected P values in the upper triangle, and uncorrected P values in the lower triangle. Notes: Age: age at diagnosis, MYCN: MYCN mutation status, Risk: high-risk, Stage: INSS tumor stage, Prog: progression, Death: death from disease

**Reviewer comment:** Clinical data and confounding: What other clinical data besides clinical outcomes used in this study are available within CAMDA 2017 dataset? There is a mention of ‘gender’ in Discussion, related to getting predictions for gender and high risk easier than for other two outcomes. In that respect - did authors checked for possible confounding between other clinical data and clinical outcomes (e.g. gender and high-risk or other outcomes) ?

Author’s response: *The clinical descriptors available are gender, age at diagnosis, MYCN mutation status and INSS tumor stage (besides progression, death from disease and high-risk). We have performed Chi-squared tests to assess the independence of these factors (see results in Table 2 below). Most of the descriptors are indeed not independent but all relationships make sense clinically and have been investigated before. For instance, late diagnosis and larger tumors are associated with poorer clinical outcomes in many cancers. This stands as well in this neuroblastoma dataset. A specificity of neuroblastoma is the influence of the mutation status of MYCN. We are indeed able to confirm that in this cohort, MYCN mutated samples are associated with poorer prognosis. To our knowledge, gender is the only descriptor that is expected to be independent. Our analysis indeed confirms that this is the case.*

**Reviewer comment:** Size of data and comparison of results: From the text I conclude that combined dataset (based on transcriptomics and aCGH data) is of the size 142 (due to mismatch in availability of both types of measurement over patients), while transcriptomics (2 express. Datasets) data is available for 498 patients (Discussed in Classification algorithms section). Figure 3B compares models from 2 sources (transcriptomics) and 3 sources (including aCGH data). According to the authors the number of patients used in these experiments is largely different? The conclusion in the text is that adding aCGH - lowers predictive power of classifier models. If there are different number of samples used in these two models - this conclusion seems flawed?

Author’s response: *We have rephrased several sentences to clarify that all comparisons were made using the same cohort (i.e., either the large cohort with 498 samples when using only transcriptomic data or the small cohort with 142 samples otherwise). In particular, the decrease in classification performance when adding genomic data is indeed observed when using only the 142 samples with genomic data.*

**Reviewer comment:** Majority voting: Authors use majority voting to combine classification models based on different genomic data or topological models. The use of majority voting in combining models is most probably not a good choice: it does not give the best results in combining models of different performances (something authors comment themselves in the text!), and it does not exploit complementarity between models. Simple weighted voting or scoring combination schemes should be a notable improvement over majority voting.

Author’s response: *Following this suggestion, we have implemented a weighted voting scheme. The weights are proportional to the performance of the respective models and have been normalized to sum up to one. The text has been updated accordingly. We thank the reviewer for this suggestion that has improved our method and the associated results.*

**Reviewer comment:** Complementarity of approaches: With improved model combination schemes authors should be able to give better answer whether different data sources (3) and representations combined together are really complementary, something that was not shown through experiments in this work. Authors also did not try to combine all representations (network and original) together to see possible complementarity.

Author’s response: *We did combine the networks and original representations together. Results were presented under the tag ‘Integrated’. We have nonetheless rephrased several sentences describing the experiments to clarify (a summary table was also introduced). In the revised version, the performance of the ‘Integrated’ models is still very much in the same range than the performance of ‘Topological’ models. They however both performed better than ‘Classical’ models. Similarly, the integration of raw genomic data (aCGH) does not result in more accurate models - a problem that was also reported by other CAMDA participants, which we now discussed more extensively (references [10, 11]).*

**Reviewer comment:** In the text (classification algorithms) mention using LDA, RF, SVM in their experiments. But, what classifier is used (and with what parameters - or how are parameters optimized?) in experiments which results are presented in Figs. 2 and 3 (also in Figures S1-S5)

Author’s response: *The objective of our study is to compare the effectiveness of the topological features regardless of the machine learning strategy. It is inspired by the original analysis of the transcriptomic data (reference [7]), in which different algorithms and strategies were used to compare the effectiveness of the microarray and RNA-seq datasets. This means that we considered the classification algorithm as a parameter, with three possible values: LDA, RF and SVM. In particular, we never selected the best models based on their performance. As a consequence, this means that the results in figures 2, 3, S1-S5 have been obtained by all algorithms (except for panels C and D of Figure S5 in which the influence of the algorithms and their parameters is reported). One group, represented as a boxplot, will always contain the three kinds of models (LDA, RF and SVM), while each point used to represent a single model (so either LDA or RF or SVM). However, points have been removed from the figures in the current version (for clarity). Similarly to the reference study ([7]), we repeated 5-fold cross-validation 10 times on the training set to get an unbiased estimate of the real performance. Regarding the parameter optimization, only the SVM parameters c (linear and radial) and gamma (radial) were optimized. In addition, SVM models were trained with default parameters for comparison purposes. We observe that parameter optimization has little to no effect. For LDA and RF, we did not identify parameters that would require to be optimized. Let us stress once again that our objective is not to identify the Şbest modelŤ but rather to investigate the usefulness of topological features, regardless of the other modeling settings. We have adapted the manuscript to highlight these points.*

**Reviewer comment:** RNA-Seq part of the CAMDA dataset (one of the two transcriptomics measurements) is first mentioned in a supplementary material (?) - which is kind of confusing. I would suggest proper and complete description of the datasets used, in the article.

Author’s response: *The two transcriptomic datasets were both introduced in the ‘Methods’ section (page 8, lines 13-15). We have nonetheless rephrased this sentence to clarify.*

**Reviewer comment:** Figure 1 is missing one step in the process - feature selection!?

Author’s response: *The legend of Figure 1 explicitly mentions that the first step is to apply dimension reduction. We have altered the figure to explicitly illustrate that feature selection takes place.*

**Reviewer comment:** Scales for balanced accuracy in figures should be made the same over all figures, in order to make easier comparison between figures.

Author’s response: *We thank the reviewer for this suggestion. We now use the same scale over all main and supplementary figures.*

**Reviewer comment:** What are the points in Figures showing performance of different models representing?

Author’s response: *The points represented the performance of the individual models and the boxplots represented the overall distributions among a group of models that share some characteristics. We have removed the points from the figure to ease reading (because we have more models and there would therefore be too many points on the figures).*

### Reviewer’s report 3: Isabel Nepomuceno

**Reviewer comment:** The analysis made by authors considers several classification scenarios by varying the number of data sources, networks and feature sets. Authors should add a table of strategies (or a paragraph in Results section) where different scenarios and settings are summarized together with the number of features that are analysed in each scenario. Reading the results section and observing Figs. 1 and 2 is a bit difficult to follow all the options under study.

Author’s response: *We thank the reviewer for this suggestion. We have added a table that summarizes all configurations (Table 1). We hope that it helps to better understand the experiments and associated results.*

**Reviewer comment:** In section Conclusions, authors claim that the network-based model and state of the art models are performing similarly, even when the network-based models are trained with far less features. However, it could be interesting to analyse if this observation holds if a feature selection algorithm is applied to the input dataset in the classical models. If this is not implemented, at least it should be mentioned as a future work in the paper.

Author’s response: *Actually, feature selection was performed first regardless of whether the selected features would be used for the classical or network-based models. We hope that the addition of Table 1 and the modification of Figure 1 clarify this.*

**Reviewer comment:** In the subsection network inference the weighted correlation network analysis (WCNA) is used. Authors should discuss why they used this method and not the classical Pearson correlation-based method. I suppose that setting the cut-off of the correlation is a difficult task and the WCNA is a ‘soft’ thresholding method that resolves this problem. Finally, an extension of this work could be to explore the hypothesis using other methods to infer gene networks using full conditional models as Markov networks or low-order conditional models.

Author’s response: *We have clarified the text to mention that two network inference methods are used concurrently, therefore creating two networks from a single data matrix. The first method is purely based on correlation and produces a fully connected network (i.e., no thresholding takes place). The second one rescales these correlation coefficients, using a soft thresholding method inspired by WGCNA. Notice that we do not try to select the best inference technique and therefore always include both networks are in all comparisons. We have also extended the conclusion to mention several avenues for future work.*

**Reviewer comment:** In the subsection Network topology, authors set p and q to 1 and 4 respectively. The meaning of the parameters p and q is not explained.

Author’s response: *The text has been updated with the full names and the effects of these two parameters.*

**Reviewer comment:** The classification algorithms used are LDA, RF and SVM. It would be interesting to include a discussion about why these algorithms were chosen and not others. For instance, one could think of using other ensemble algorithm like gradient boosting machine (XGBoost is the most known implementation). This is very popular because over half of the winning solutions for the data science competition Kaggle in 2015 contain XGBoost. Among the 29 challenge winning solutions 17 solutions used XGBoost (1). I’m not asking the authors to include a new experimentation with this algorithm, but to discuss a little bit about their choice. (1) Chen T, Guestrin C. XGBoost: A Scalable Tree Boosting System. arXiv:160302754 [cs]. 2016;785–94.

Author’s response: *The algorithm selection was based on the previous extensive analysis of this neuroblastoma dataset (reference [7]). In particular, we selected the algorithms producing most frequently the best results (as described in the supplementary file of [7]).*

**Reviewer comment:** In the legend of Suplemmentary Figure 1 authors should explain that only transcriptomic data are used instead of combine genomic data as in Figure 5 is mentioned. I supposed it after reading the first paragraph of section Results.

Author’s response: *The legends of all figures have been updated to clarify exactly which cohort has been used (large when all 498 samples have been used - small when only the 142 samples with genomic data have been used). Table 1 also summarizes relevant information for all experiments.*

**Reviewer comment:** In second paragraph of section Results, the performance of topological against full ltopological model is compared, (Figures 2B and 3C is explained). Authors should detail which of the three network derived feature sets have been used.

Author’s response: *All feature sets were used. This has been clarified in the text.*

## Additional files

Additional file 1 Supplementary **Figures S1-S5** and **Tables S1-S2**. (PDF 1266 kb)

Additional file 2 Impact of *node2vec* parameters on the feature vectors. This file describes a preliminary analysis to better understand the effect of the different *node2vec* parameters on the produced feature vectors. (PDF 1082 kb)

Additional file 3 aCGH data processing. This file describes the processing of the aCGH dataset. (PDF 1773 kb)

## Abbreviations

ACC: Accuracy
aCGH: Array Comparative Genomic Hybridization
bACC: Balanced accuracy
CAMDA: Critical Assessment of Massive Data Analysis
GEO: Gene Expression Omnibus
LDA: Linear Discriminant Analysis
MCC: Matthews Correlation Coefficient
PCA: Principal Component Analysis
PSN: Patient Similarity Networks
RF: Random Forest
RNA: RiboNucleic Acid
SBM: Stochastic Block Model
SNF: Similarity Network Fusion
SVM: Support Vector Machine
TCGA: The Cancer Genome Atlas
WGCNA: Weighted Correlation Network Analysis
