# Supplementary material for: Predicting clinical outcome of neuroblastoma patients using an integrative network-based approach
Source: Biol Direct. 2018 Jun 7;13:12. doi: 10.1186/s13062-018-0214-9 (PMC5992838; doi:10.1186/s13062-018-0214-9)
Supplement: Supplementary file 1 — Supplementary Figures S1-S5 and Tables S1-S2. (PDF 1266 kb) [file 13062_2018_214_MOESM1_ESM.pdf]

Additional file 1 for "Predicting clinical outcome of neuroblastoma patients using an integrative network-based approach"

Léon-Charles Tranchevent[1], Petr V. Nazarov[1], Tony Kaoma[1], Georges P. Schmartz[1,2], Arnaud Muller[1], Sang-Yoon Kim[1], Jagath C. Rajapakse[3], and Francisco Azuaje[1]

[1]Proteome and Genome Research Unit, Department of Oncology, Luxembourg Institute of Health, Luxembourg.
[2]Bioinformatics bachelor program, Universität des Saarlandes, Saarbrücken, Germany.
[3]Bioinformatics Research Center, School of Computer Engineering, Nanyang Technological University, Singapore

List of Figures

S1 Performance of the components of the topological models on the small cohort. . . . 2
S2 Detailed performance of the models using centralities. 2
S3 Performance of the classical models using various data sources. 3
S4 Performance of SNF based topological models. 3
S5 Impact of classification algorithms and network-based parameters on performance. 4

List of Tables

S1 Comparison of the different modeling strategies 5
S2 Influence of the data sources on model performance 6

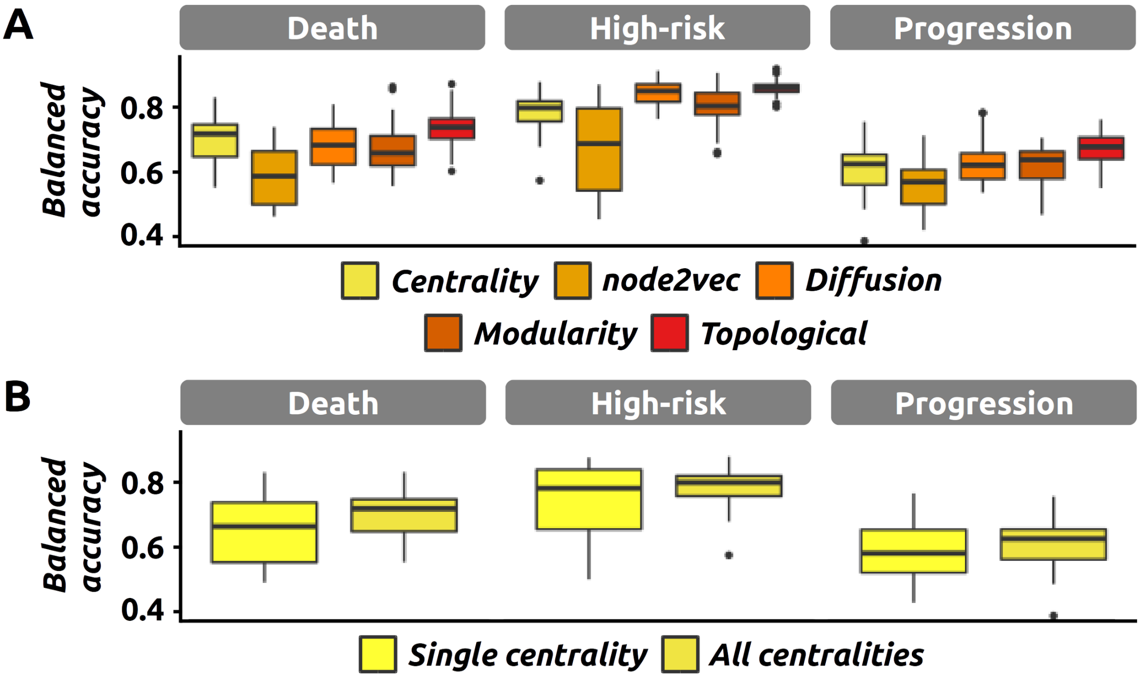

Supplementary Figure S1: The performance (*i.e.*, balanced accuracy) of classification models in various settings, and for the three clinical endpoints of interest. The results presented in this figure were obtained on the small cohort. (A) Performance of models using only one of the four feature sets at once (Centrality, node2vec, diffusion and modularity) or all of them. (D) Performance of models using a single centrality metric or all centrality metrics at once. Note: results for the large cohort are presented in Figure 2C-D.

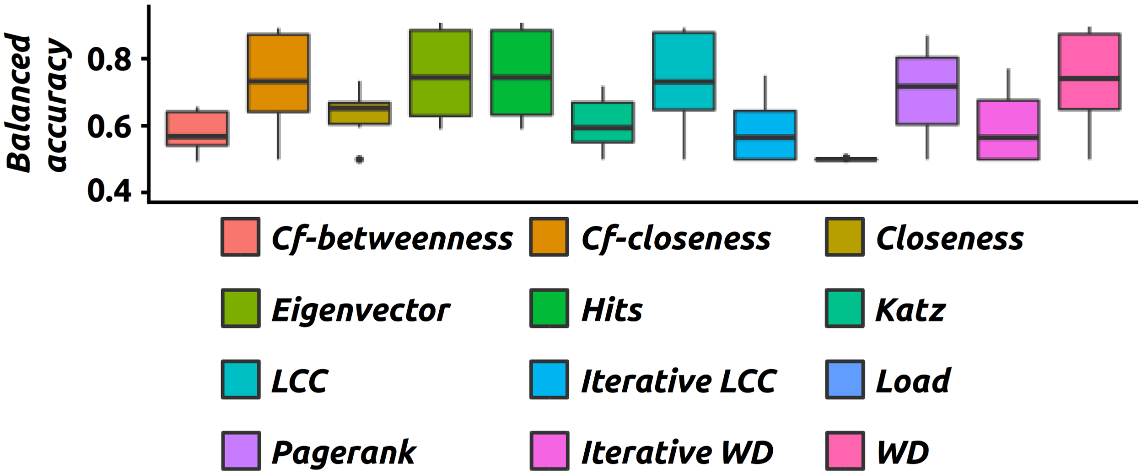

Supplementary Figure S2: The performance (*i.e.*, balanced accuracy) of classification models using a single centrality metric, regardless of the clinical endpoints. The results have been obtained on the large cohort. [Cf: Current-flow - LCC: Local Clustering Coefficient - WD: Weighted Degree]

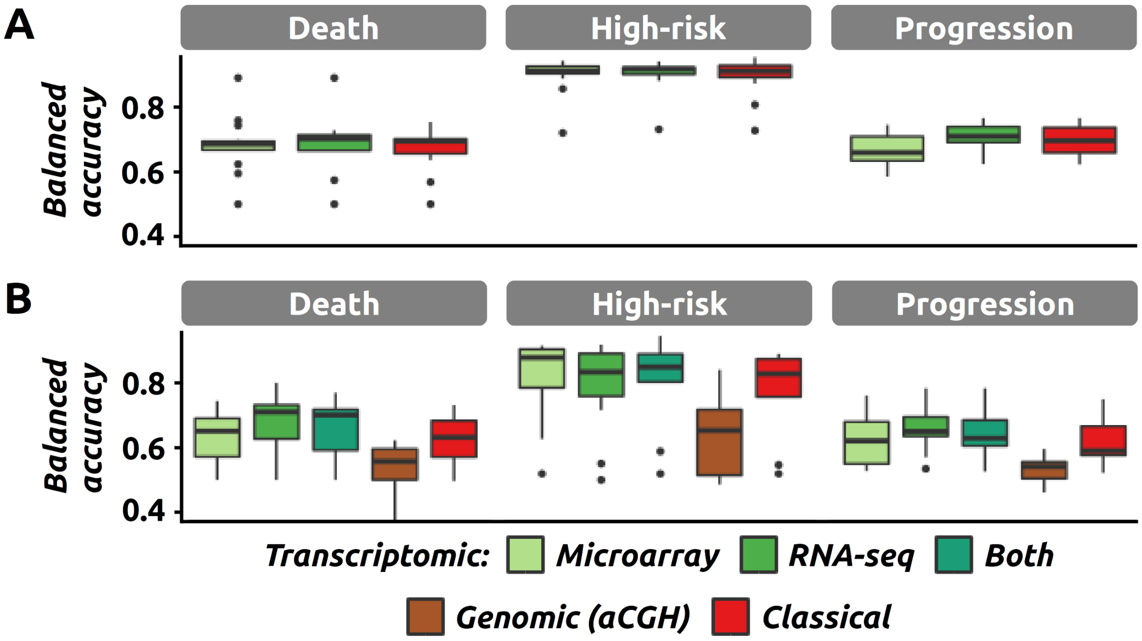

Supplementary Figure S3: The performance (*i.e.*, balanced accuracy) of classical models in various settings, and for the three clinical endpoints of interest. (A) Performance of the classical models relying only on a single transcriptomic data source (greens), or on both sources. Results were obtained on the large cohort. (B) Same as A but on the small cohort. Performance of topological models using one (greens and maroon), two (dark green, only transcriptomic) or three data sources (red). Note: Results for topological models are presented in Figure 3.

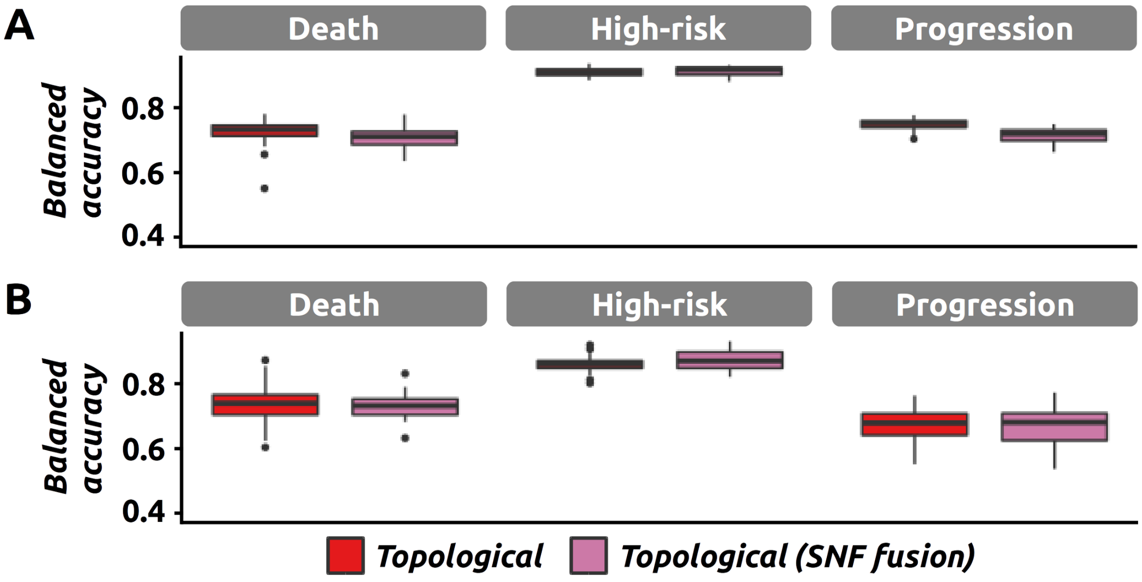

Supplementary Figure S4: The performance (*i.e.*, balanced accuracy) of various topological models for the three clinical endpoints of interests. (A) Performance of topological models using the two transcriptomic data sources as introduced in the manuscript (topological) or using SNF to fuse the two networks prior to feature extraction. Results were obtained on the large cohort. (B) Same as A but on the small cohort (with three networks instead of two).[SNF: Similarity Network Fusion]

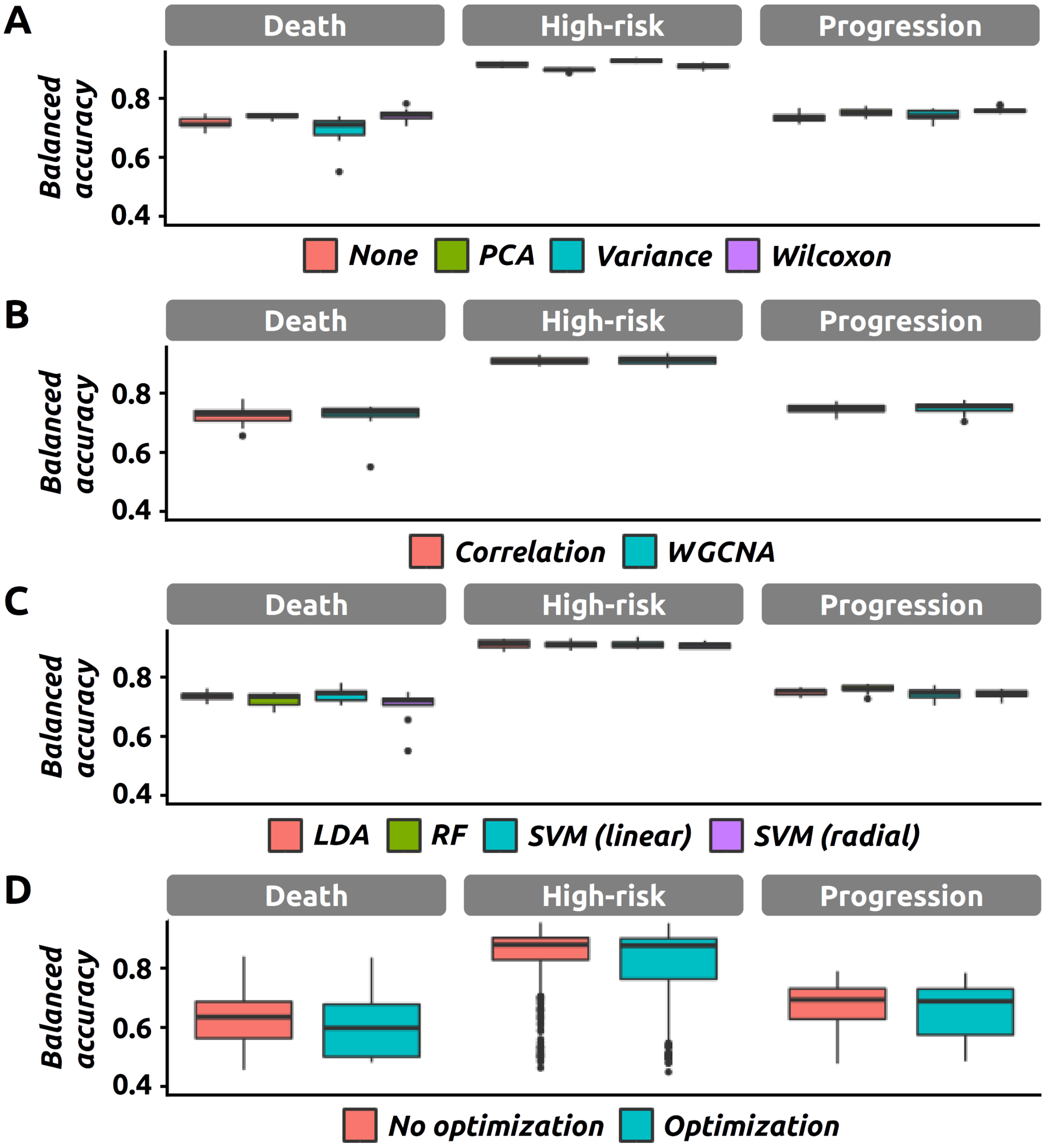

Supplementary Figure S5: The performance (*i.e.*, balanced accuracy) of various classification models for the three clinical endpoints of interest. Results were obtained on the large cohort using only topological models (A-C) or all models (D). (A) Performance of topological models using different dimensionality reduction strategies. (B) Performance of topological models using different network inference strategies. (C) Performance of topological models using different classification algorithms. (D) Performance of all kind of models using SVM algorithms with their default parameters (No optimization) or with optimal parameters defined through cross-validation (Optimization). [None: models based on all omics features - PCA: models based on pseudo-features built through PCA - Variance: models based on a selection of the most varying features - Wilcoxon: models using a Wilcoxon-based strategy to select omics features].

Experiment	Death from disease		High-risk		Progression	
	p-value	Δ_{bACC}	p-value	Δ_{bACC}	p-value	Δ_{bACC}
Topological vs classical						
Large cohort	7e-4	5%	0.35	1%	<1e-7	5%
Small cohort	4e-6	12%	2e-3	8%	6e-3	6%
Integrated vs classical						
Large cohort	4e-3	5%	0.23	2%	3e-3	3%
Small cohort	7e-3	8%	6e-3	8%	0.81	1%
Topological vs integrated						
Large cohort	0.99	0%	0.83	0%	0.05	2%
Small cohort	0.24	4%	0.94	0%	0.04	5%
Centrality vs single centrality						
Large cohort	1e-7	8%	1e-7	12%	9e-6	7%
Small cohort	0.02	5%	0.12	4%	0.34	2%
Topological vs fused						
Large cohort	0.03	2%	0.29	0%	<1e-7	3%
Small cohort	0.63	0%	0.03	-2%	0.51	1%
Centrality vs classical						
Large cohort	0.51	1%	0.19	1%	0.09	-2%

Supplementary Table S1: Comparison of classical, topological, integrated and fused models using both cohorts. For each comparison, the reported values are (i) a p-value obtained by a one way ANOVA followed by a post-hoc Tukey test and (ii) the accuracy gain Δ_{bACC}, computed as the difference between the averages of the two groups.

Experiment	Death from disease p-value	Death from disease Δ_{bACC}	High-risk p-value	High-risk Δ_{bACC}	Progression p-value	Progression Δ_{bACC}
[Topological] Transcriptomic (both) vs transcriptomic (microarray)						
Large cohort	0.39	0%	1e-3	1%	0.07	1%
Small cohort	0.86	1%	1	0%	0.73	1%
[Topological] Transcriptomic (both) vs transcriptomic (RNA-seq)						
Large cohort	0.53	0%	1	0%	0.72	0%
Small cohort	0.99	0%	0.90	-1%	1	0%
[Classical] Transcriptomic (both) vs transcriptomic (microarray)						
Large cohort	0.92	-1%	0.98	0%	0.27	3%
Small cohort	0.90	3%	1	-1%	0.94	2%
[Classical] Transcriptomic (both) vs transcriptomic (RNA-seq)						
Large cohort	0.81	-2%	0.96	-1%	0.59	-2%
Small cohort	0.99	-2%	1	2%	0.97	-2%
[Small cohort] All three sources[a] vs transcriptomic (both)						
Topological	3e-4	-6%	0.48	-2%	1e-4	-5%
Classical	0.67	-5%	0.99	-3%	0.86	-3%
[Small cohort] Genomic (aCGH) vs transcriptomic (microarray)						
Topological	<1e-7	-21%	<1e-7	-12%	<1e-7	-13%
Classical	0.08	-9%	0.01	-18%	0.01	-9%
[Small cohort] Genomic (aCGH) vs transcriptomic (RNA-seq)						
Topological	<1e-7	-23%	<1e-7	-14%	<1e-7	-14%
Classical	2e-4	-14%	0.04	-15%	2e-4	-13%

Supplementary Table S2: Comparison of models relying on different data sources using both topological and classical models and both cohorts. For each comparison, the reported values are (i) a p-value obtained by a one way ANOVA followed by a post-hoc Tukey test and (ii) the accuracy gain Δ_{bACC}, computed as the difference between the averages of the two groups. [a] Equivalent respectively to topological and classical models in Table 1.
