# Supplementary material for: Predicting clinical outcome of neuroblastoma patients using an integrative network-based approach
Source: Biol Direct. 2018 Jun 7;13:12. doi: 10.1186/s13062-018-0214-9 (PMC5992838; doi:10.1186/s13062-018-0214-9)
Supplement: Supplementary file 2 — Impact of node2vec parameters on the feature vectors. This file describes a preliminary analysis to better understand the effect of the different node2vec parameters on the produced feature vectors. (PDF 1082 kb) [file 13062_2018_214_MOESM2_ESM.pdf]

Additional file 2 for "Predicting clinical outcome of neuroblastoma patients using an integrative network-based approach"

Léon-Charles Tranchevent[1], Petr V. Nazarov[1], Tony Kaoma[1], Georges P. Schmartz[1,2], Arnaud Muller[1], Sang-Yoon Kim[1], Jagath C. Rajapakse[3], and Francisco Azuaje[1]

[1]Proteome and Genome Research Unit, Department of Oncology, Luxembourg Institute of Health, Luxembourg.
[2]Bioinformatics bachelor program, Universität des Saarlandes, Saarbrücken, Germany.
[3]Bioinformatics Research Center, School of Computer Engineering, Nanyang Technological University, Singapore

1 Global processing workflow

For aCGH, we extracted the 185 samples, corresponding to 145 patients for which we also had expression data. To account for the fact that the aCGH data were produced using different technologies, the profiles were filtered to keep only the genomic features that are shared by all platforms. A few samples were discarded in the process: three samples because of the low amount of shared genomic features with others platforms and one sample because the corresponding platform was used only once, which is suboptimal for an efficient batch effect correction.

After this step, we had 181 samples left, for 142 patients, with 39 patients associated with two replicates. A correlation analysis revealed that 22 of these samples exhibited a negative correlation with their respective replicates (correlation $<$ -0.9), while the remaining samples correlated positively with their respective replicates (correlation $>$ 0.9). This indicated a possible inversion of the signal in a selection of the samples. A hierarchical clustering analysis indicated which samples were most likely reversed and identified in addition another 8 samples (without replicates) with similar profiles. The signal of 30 aCGH samples was therefore inverted to correct for these potential errors (see more details in section 2).

Since the aCGH data were produced by different laboratories and using different arrays, the data was further normalized to correct for the potential lab, platform and batch effects. For the 39 samples with aCGH replicates, the signal was averaged over the replicates. This is motivated by the fact that the correlation between the replicates was very high ($>$ 0.9), when compared to correlations between non replicates ($<$ 0.85).

2 Error correction

This section summarizes the effort undertaken to correct potential errors in the aCGH dataset. This was performed by a manual inspection of the data using a correlation analysis in R. For a more accurate analysis, only the aCGH signal from the X chromosome was used. Notice that we were not able to discriminate patients based on their gender using the sampled data (data not shown).

We started by investigating the 39 aCGH samples with replicates since 22 of these surprisingly exhibit a strong negative correlation (see Figure 1-Left). The figure shows that there are two clusters, with one composed exclusively of potentially problematic samples (bottom-right cluster). We reverted the signal of these 22 samples and observed a much more homogeneous heatmap after correction (see Figure 1-Right). In particular, the samples that have been corrected do not cluster together any more.

We extended the analysis by considering the samples without replicates (*hereinafter* single), for which it is therefore not possible to get a hint about a possible error. We started by plotting

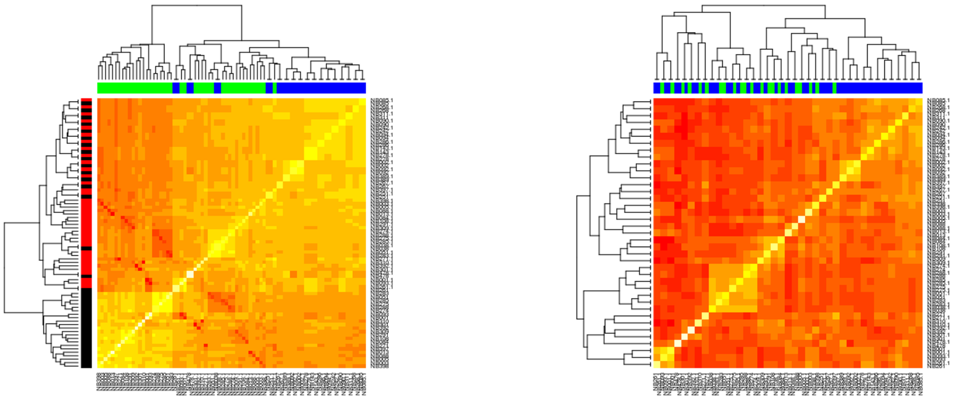

Figure 1: (Left) Heatmap based on sample correlation. Only samples with replicates are shown. On the y axis, red means first sample, black means replicate sample. On the x-axis, blue means that the correlation with the replicate is fine (> 0.9), green means that the correlation with the replicate is very low < -0.9). (Right) Heatmap similar to 'Left', but after correction. Green samples are the 22 samples that have been corrected.

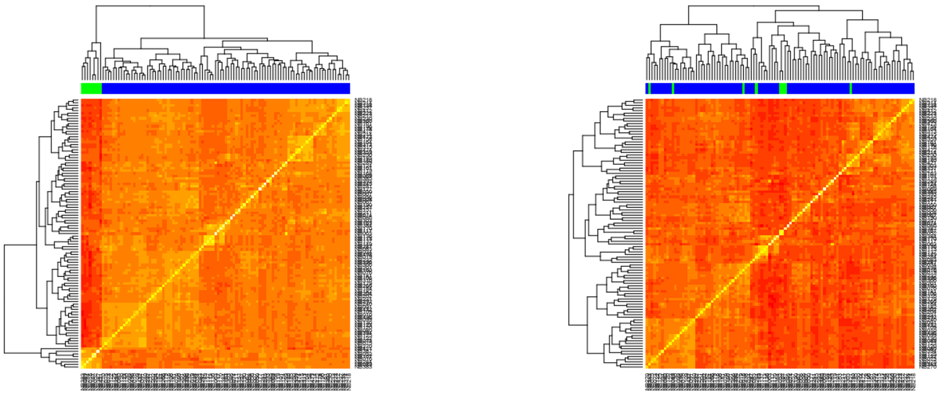

Figure 2: (Left) Heatmap based on sample correlation. Only single samples are shown. Green / blue are the two main clusters identified. (Right) Heatmap similar to 'Left', but after correction. Green samples are the 8 samples that have been corrected.

the heatmap for all single samples (see Figure 2-Left). As can be appreciated from the figures, we once again observed two groups of samples. The smallest group (bottom-right) also appears to cluster with the 22 already identified problematic samples (data not shown). After correction, we observe again a much more homogeneous heatmap, and the corrected samples do not cluster together any more (see Figure 2-Right).

To conclude, we have corrected 30 aCGH samples by inverting the raw signal. The full list is present in table 1. We also noticed that our list and the list of the samples corrected by another CAMDA team are very different [1].

References

[1] Suo C., Deng W., Nghia Vu T., Shi L., Pawitan Y.: *Accumulation of Potential Driver Genes with Genomic Alterations Predicts Survival in High-Risk Neuroblastoma.*, in Proceedings of the CAMDA conference (2017)

Sample id
NB383
NB284
NB089
NB016
NB421
NB387
NB167
NB052
NB398
NB003
NB005
NB088
NB013
NB397
NB271
NB084
NB108
NB291
NB309
NB392
NB301
NB310
NB001
NB093
NB274
NB288
NB275
NB285
NB051
NB283

Table 1: List of potentially problematic aCGH samples.
