# Supplementary material for: Predicting clinical outcome of neuroblastoma patients using an integrative network-based approach
Source: Biol Direct. 2018 Jun 7;13:12. doi: 10.1186/s13062-018-0214-9 (PMC5992838; doi:10.1186/s13062-018-0214-9)
Supplement: Supplementary file 3 — aCGH data processing. This file describes the processing of the aCGH dataset. (PDF 1773 kb) [file 13062_2018_214_MOESM3_ESM.pdf]

Additional file 3 for "Predicting clinical outcome of neuroblastoma patients using an integrative network-based approach"

Léon-Charles Tranchevent[1], Petr V. Nazarov[1], Tony Kaoma[1], Georges P. Schmartz[1,2], Arnaud Muller[1], Sang-Yoon Kim[1], Jagath C. Rajapakse[3], and Francisco Azuaje[1]

[1]Proteome and Genome Research Unit, Department of Oncology, Luxembourg Institute of Health, Luxembourg.
[2]Bioinformatics bachelor program, Universität des Saarlandes, Saarbrücken, Germany.
[3]Bioinformatics Research Center, School of Computer Engineering, Nanyang Technological University, Singapore

In order to investigate the effect of the *node2vec* parameters on the features vectors produced by *node2vec*, we have performed a small experiment in which a single parameter is explored using a small grid search while all other parameters are kept to their default values. We then report the distribution of all values produced by node2vec, the correlation between the produced vectors and the correlation between the analyzed samples. This analysis was performed on the CAMDA 2017 challenge transcriptomic data (microarray and RNA-seq) using R and ggplot2.

We first study the parameter d that controls the length of the produced feature vectors. Its default value is 128. Our search space went from 32 to 1024, increasing by power of 2. The results indicate that the range of the produced values is reduced when d increases (see Figure 1). In addition, we can observe a trend between the number of features and their correlation. It seems that producing more features increases the likelihood of producing highly correlated (or anti-correlated features) (see Figure 2). It is important to notice however that this is not an exact rule as the number of highly correlated / anti-correlated features can go up and down when d increases (see Figure 2-Left). Also, there does not seem to be a general rule about which value of d gives the smaller number of highly correlated / anti-correlated features, even when the original data approximately has the same size. The influence on the correlation between sample pairs is smaller to some extend (see Figure 3). It is important to notice that all correlations are extremely high (> 0.9), reflecting the moderate ability of these features to discriminate between patients with different clinical features.

We then study a second parameter r that controls the number of random walks per source. Its

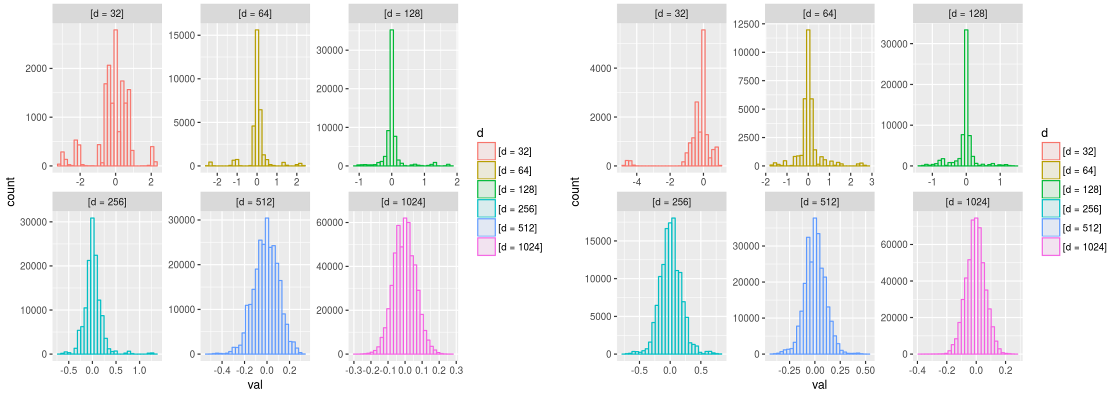

Figure 1: Histograms of the produced values (feature vectors) for different values of parameter d. (Left) Microarray data. (Right) RNA-seq data.

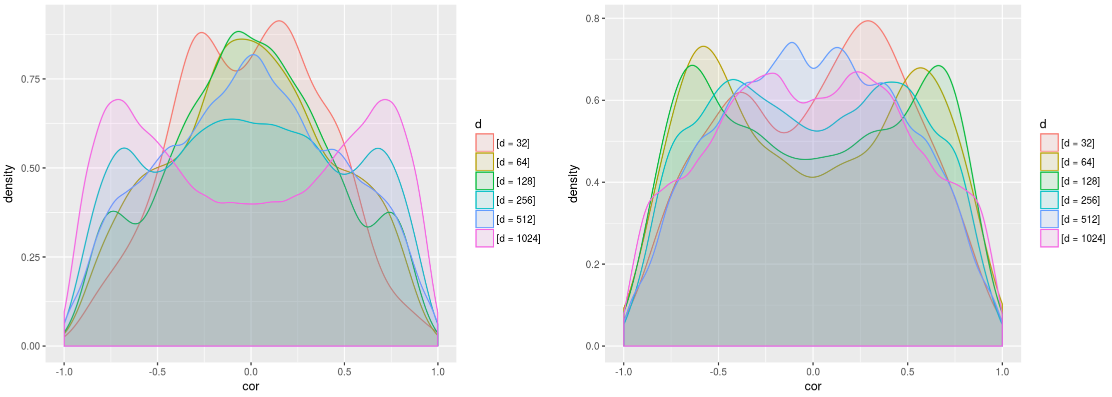

Figure 2: Histograms of the correlation between pairs of feature vectors for different values of parameter d. (Left) Microarray data. (Right) RNA-seq data.

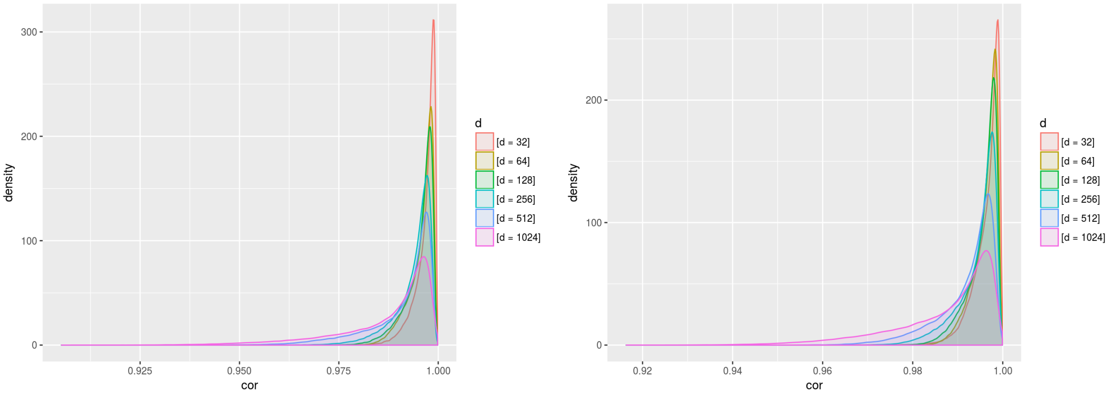

Figure 3: Histograms of the correlation between pairs of samples for different values of parameter d. (Left) Microarray data. (Right) RNA-seq data.

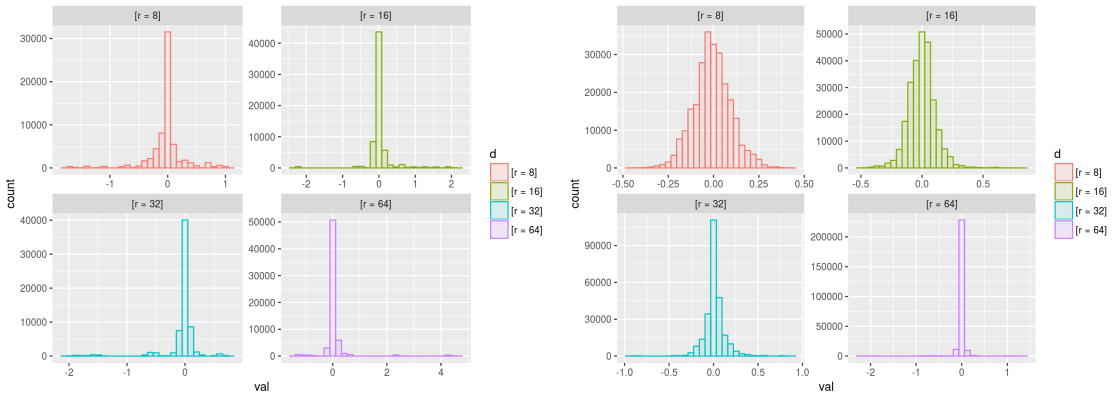

Figure 4: Histograms of the produced values (feature vectors) for different values of parameter r. (Left) Microarray data. (Right) RNA-seq data.

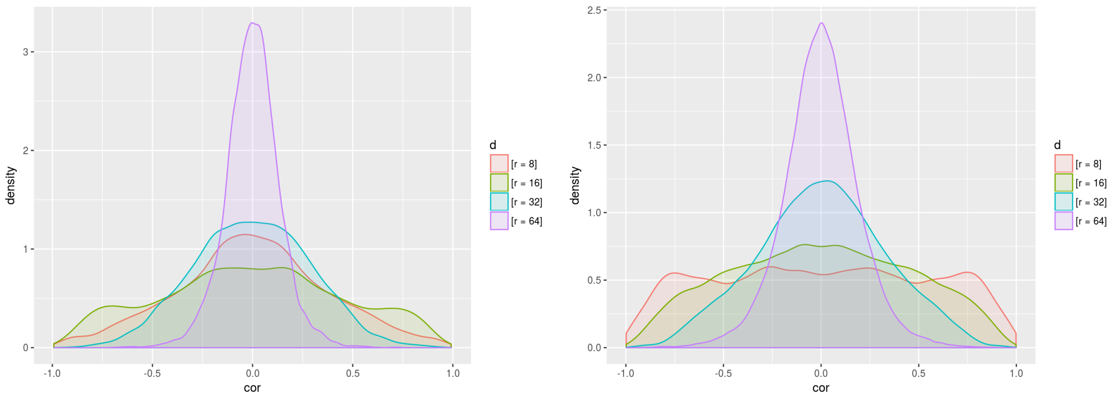

Figure 5: Histograms of the correlation between pairs of feature vectors for different values of parameter r. (Left) Microarray data. (Right) RNA-seq data.

default value is 10. Our search space went from 8 to 64, increasing by power of 2 once again. The effect on the ranges and distributions of produced values is unclear but at least different fort the two studied datasets (see Figure 5). Similar to the d parameter, we can observe a trend between the number of random walks per source and the feature vector correlation. It seems that exploring more walks decreases the likelihood of producing highly correlated (or anti-correlated features) (see Figure 5). It is important to notice however that this is not an exact rule as the number of highly correlated / anti-correlated features can go up and down when r increases (see $r = 8$ and $r = 16$ in Figure 5). Once again, there does not seem to be a general rule about which value of r gives the smaller number of highly correlated / anti-correlated features, even when the original data approximately has the same size. We observe a similar influence on the correlation between sample pairs (see Figure 6). The higher the number of walks, the more evenly distributed are the correlations. However, this is once again only a trend, not a general rule (see $r = 32$ and $r = 64$ in Figure 6). It is important to notice that all correlations are extremely high (> 0.9), reflecting once again the moderate ability of these features to discriminate between patients with different clinical features.

The effect of these two parameters on the produced feature vectors is unclear, and it seems difficult to select optimal parameters since even small adaptations can completely change the vectors, and therefore their ability to classify patients into clinically relevant groups. This can be due to the complex nature of the problem at hand. For instance, varying the parameter d will either decrease the correlation between samples in general, or decrease the correlation between feature vectors, but not both at the same time (which would be desirable), at least not in all cases.

We have used all these configurations for the CAMDA 2017 prediction challenge. We do not observe a clear trend between the performance (measured with the balanced accuracy) and any of the two parameters. For RNA-seq, the performance when varying the parameter d even looks cyclic (back and forth between 0.4 to 0.7).

To conclude, this is only a preliminary study that would need to be extended to really under-

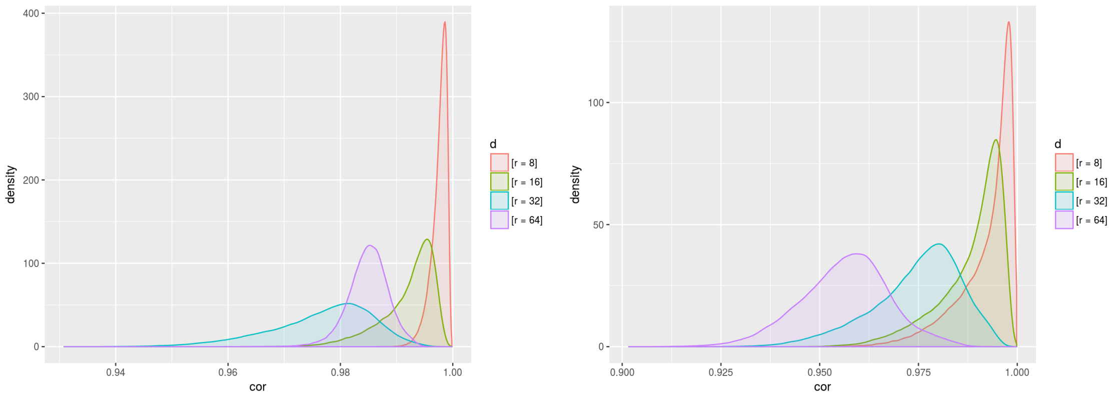

Figure 6: Histograms of the correlation between pairs of samples for different values of parameter r. (Left) Microarray data. (Right) RNA-seq data.

stand how to make the best of the *node2vec* software. This could be done by extending the grid search and performing a full validation.
